# Supplementary material for: Acknowledging the impact of seasonal blood pressure variation in hypertensive CKD and non-CKD patients living in a Mediterranean climate
Source: PLoS One. 2023 Dec 7;18(12):e0293403. doi: 10.1371/journal.pone.0293403 (PMC10703340; doi:10.1371/journal.pone.0293403)
Supplement: S1 Fig — (DOCX) [file pone.0293403.s001.docx]

**S1 Figure. Flow diagram of patients' inclusion**
