# Supplementary material for: Acknowledging the impact of seasonal blood pressure variation in hypertensive CKD and non-CKD patients living in a Mediterranean climate
Source: PLoS One. 2023 Dec 7;18(12):e0293403. doi: 10.1371/journal.pone.0293403 (PMC10703340; doi:10.1371/journal.pone.0293403)
Supplement: S1 Table — (DOCX) [file pone.0293403.s002.docx]

**S1 Table. Average monthly temperatures between 2006 and 2020**

| **YEAR** | **JAN** | **FEB** | **MAR** | **APR** | **MAY** | **JUN** | **JUL** | **AUG** | **SEP** | **OCT** | **NOV** | **DEC** |
| --- | --- | --- | --- | --- | --- | --- | --- | --- | --- | --- | --- | --- |
| **2006** | 8.1 | 9.9 | 12.2 | 15.9 | 19.6 | 23.6 | 25 | 26.3 | 23.9 | 19.8 | 12.5 | 8.2 |
| **2007** | 7.7 | 9.7 | 11.5 | 14.1 | 20.7 | 23.7 | 26.3 | 26.1 | 24 | 21.2 | 14.8 | 9.3 |
| **2008** | 5.4 | 8.3 | 15 | 17.7 | 18.6 | 24.2 | 26 | 26.8 | 24.4 | 20 | 15 | 9.7 |
| **2009** | 8.4 | 9.8 | 11 | 15.6 | 19.4 | 24.4 | 26 | 26.2 | 23.1 | 21.7 | 13.8 | 11.7 |
| **2010** | 10.9 | 11.2 | 14.2 | 16.7 | 20.4 | 23.9 | 26.1 | 28.1 | 25.5 | 22.1 | 16.8 | 11.7 |
| **2011** | 9.5 | 9.8 | 11.7 | 15.2 | 18.9 | 22.9 | 26.4 | 26.2 | 24.3 | 19.4 | 11 | 8.9 |
| **2012** | 7.7 | 7.7 | 9.9 | 16.9 | 20.3 | 24.8 | 27.1 | 26.6 | 24.9 | 21.3 | 16.1 | 10.8 |
| **2013** | 9.1 | 11.3 | 14 | 16.1 | 21 | 23.4 | 25.2 | 25.9 | 23.3 | 18.7 | 16.4 | 8.3 |
| **2014** | 9.9 | 10.7 | 13.4 | 17.2 | 19.9 | 22.9 | 25.6 | 26.7 | 23.3 | 19.7 | 13.6 | 11.7 |
| **2015** | 8.2 | 9.5 | 12.9 | 14.4 | 20.2 | 22 | 25.7 | 26 | 25.6 | 21.3 | 15.1 | 8.6 |
| **2016** | 7.7 | 12 | 13.4 | 18.3 | 19.8 | 24.3 | 26.3 | 27.2 | 23.4 | 21.1 | 14.4 | 7.6 |
| **2017** | 7 | 7.8 | 12.4 | 15.9 | 19.9 | 23.7 | 26.9 | 26.7 | 25.3 | 19.6 | 14.4 | 11.9 |
| **2018** | 9.3 | 12 | 15.3 | 17.2 | 20.9 | 23.6 | 25.9 | 26.2 | 24.8 | 21 | 14.8 | 10.4 |
| **2019** | 8.4 | 10 | 11.3 | 14 | 21.4 | 24.4 | 25.4 | 26.2 | 24.3 | 21.9 | 16 | 10.5 |
| **2020** | 8.1 | 9.3 | 12.7 | 15.4 | 20.2 | 22.6 | 26.4 | 26.3 | 26.1 | 22.9 | 15 | 11.5 |
| **2021** | 17 | 16 | 16 | 19 | 23 | 25 | 28 | 29 | 27 | 25 | 22 | 17 |
| **2022** | 14 | 15 | 13 | 20 | 22 |  |  |  |  |  |  |  |
| Climatic Research Unit Country File created on Mon 8 Mar 13:29:59 GMT 2021, from CRU TS run #2103051243 Country = Lebanon: parameter = Mean Temperature: Units = degrees Celsius |  |  |  |  |  |  |  |  |  |  |  |  |
|  |  |  |  |  |  |  |  |  |  |  |  |  |
|  |  |  |  |  |  |  |  |  |  |  |  |  |
