# Supplementary material for: Acknowledging the impact of seasonal blood pressure variation in hypertensive CKD and non-CKD patients living in a Mediterranean climate
Source: PLoS One. 2023 Dec 7;18(12):e0293403. doi: 10.1371/journal.pone.0293403 (PMC10703340; doi:10.1371/journal.pone.0293403)
Supplement: Attachment — Submitted filename: Review.docx [file pone.0293403.s003.docx]

Very interesting article. Just a few observations.

Page 12.

Indeed, it showed that antihypertensive dose reduction and **molecules’ removal** occurred during the warm season whereas adding treatment was predominant in the cold season.

Clarify which antihypertensive medication were implicated in this fluctuation.

Explain and clarify the use of the word “molecule”. It does not seem to fit.

Conclusions:

Blood pressure gets lower in warm seasons and higher in cold seasons in both CKD and non-CKD hypertensive patients.

Explain is this is due to hydration status.
